# The prognostic value of p53 mutation in pediatric marrow hypoplasia

**DOI:** 10.1186/1746-1596-6-58

**Published:** 2011-06-30

**Authors:** Hasnaa A Abo-Elwafa, Fadia M Attia, Alzahraa EA Sharaf

**Affiliations:** 1 Deptartment of Clinical Pathology, Faculty of Medicine, Sohag University, University Street, Sohag, Egypt; 2 Deptartment of Clinical Pathology, Faculty of Medicine, Suez Canal University, New University Street, Suez Canal, Egypt; 3 Deptartment of Pediatric, Faculty of Medicine, Sohag University, University Street, Sohag, Egypt

## Abstract

**Background:**

The tumor suppressor gene p53 is involved in the control of cell proliferation, particularly in stressed cells. p 53 gene mutations are the most frequent genetic event found in human cancers. Fanconi Anemia (FA) is the most common representative of inherited bone marrow failure syndromes (IBMFS) with a leukemic propensity. P 53 DNA alteration has not been studied before in Egyptian children with FA.

**Patients and methods:**

we investigated p53 mutation in the bone marrow and peripheral blood of forty children, FA (n = 10), acquired aplastic anemia (AAA) (n = 10), and immune thrombocytopenia (ITP) as a control (n = 20), using real-time PCR by TaqMan probe assay

**Results:**

Mutation of p53 gene was demonstrated in the BM of 90% (9/10) of children with FA, compared to 10% (1/10) in AAA (p < 0.001), while, no p53 DNA mutation was seen in the control group. A positive correlation between DNA breakage and presence of p53 mutation was seen in FA (p < 0.02, r0.81).

**Conclusion:**

mutation of p53 gene in hypoplastic marrow especially FA may represent an early indicator of significant DNA genetic alteration with cancer propensity.

## Introduction

Patients with IBMFS are at increased risk of malignant transformation, possibly due to cell cycle deregulation as marked by the over expression of cell cycle markers as p53 and Ki-67 [1,2]. Fanconi's anemia (FA), the most common disorder of IBMFS, is a rare autosomal recessive disorder (prevalence of 1-5 per million) with bone marrow failure, developmental anomalies, and predisposition to leukemia and solid tumors [3-5].

The p53 gene plays a major role in cell-cycle regulation, particularly in the presence of a genetic alteration in DNA, a critical step for the initiation of leukemogenesis [6].

In healthy humans, the p53 protein is continually produced and degraded in the cell. However, it becomes activated in response to multiple types of stresses, which include DNA damage, oxidative stress, osmotic shock, ribonucleotide depletion and deregulated oncogene expression [7]. More than 50% of human tumors contain a mutation or a deletion in the p53 gene. Increasing the expression of p53 gene, which is a tumour suppressor gene involved in the apoptotsis, and the control of cell proliferation may be a good way to treat tumors or prevent them from spreading. Thus, restoring endogenous p53 function as the use of gene therapy holds a lot of promise in future treatment of malignancies [8-10].

Since p53 is not over expressed in the BM of hematologically normal individuals [11,12]. We hypothesized that DNA damage and cancer predisposition in BM failure disorders could be related to p53 gene alterations. We studied the expression of p53 mutation, as a marker of cell cycle dysregulation, in BM of children with FA as well as children with AAA.

## Subjects and Methods

### Study design and populations

the study was approved by the Ethical Committee of Clinical Pathology Dept., and Pediatric Dept., Sohag University and Clinical Pathology Dept., Suez Canal University, during the period from April 2007 to June 2010. The study groups included 20 child (9 males and 11 females) with aplastic anemia some of them with congenital malformations (two brothers the old one is 11 years old at the time of sampling he had short stuttered, microcephally, brown skin pigmentation, the younger is 5 years old has extra thumb, girl from another family her age is 12 years, also she had short stuttered, microcephally, brown skin pigmentation and twins boys from another family, they had short stuttered, microcephally, brown skin pigmentation, one of them diagnosed AML at date of presentation ) and other 20 child with ITP (7 males and 13 females) as a control. The age of children at presentation in the study ranged from 4 to 14 years. They were visiting the Hematology Outpatient Clinic, Pediatric Department, Sohag University Hospital. Family history was recorded in pre-designed proforms and a detailed pedigree was taken in each case. Informed consent was taken from the patients or/and their parents in case of younger. All patients in the study and the control groups were subjected to clinical examination laboratory investigations including peripheral hemogram, bone marrow aspiration (BMA). Trephine bone marrow biopsy (BMB) for cases with marrow hypoplasia. Mytomycin C (MMC) stress test and detection of p53 gene mutation were done in all patients of the study and the control groups.

### Methods

All the study populations were subjected to:

-Routine investigations (random blood sugar, renal function tests and liver function testes on CX-9 Beckman - coulter., France ).

**Figure 1:**
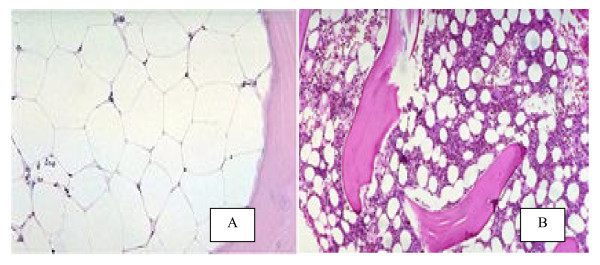
**BMB sections by (H & E staining)**: (A) Severe hypoblastic marrow: marked increase in fat space more than 80%. (B) Mild hypoblastic marrow: Just increase in fat space slight over 50%.

- Complete blood count (Cell Dyne - 2700, Abbott Lab., USA).

- **Assessment of BM cellularity**:

**BMA **samples were obtained from all patients of the study and the control groups by anterior superior iliac spine approach. The smears were stained by ordinary stains, examined for the cellular morphology. **BMB **was done to all patients with hypoplastic marrow, it performed under general anesthesia, through posterior superior iliac spine approach by Islamic biopsy needle No. 8, No. 12. Paraffin's embedded sections were examined by H & E stain for detection of marrow architecture and cellularity, reticuline stain was used to detect marrow fibrosis. BM cellularity was independently and blindly graded according to Frisch and Lewis [13]: definite hypercellularity (grade 6); moderate hypercellularity (grade 5); normal cellularity (grade 4); mild hypocellularity (grade 3); moderate hypocellularity (grade 2) and, severe hypocellularity (grade 1), Figure 1.

**Figure 2:**
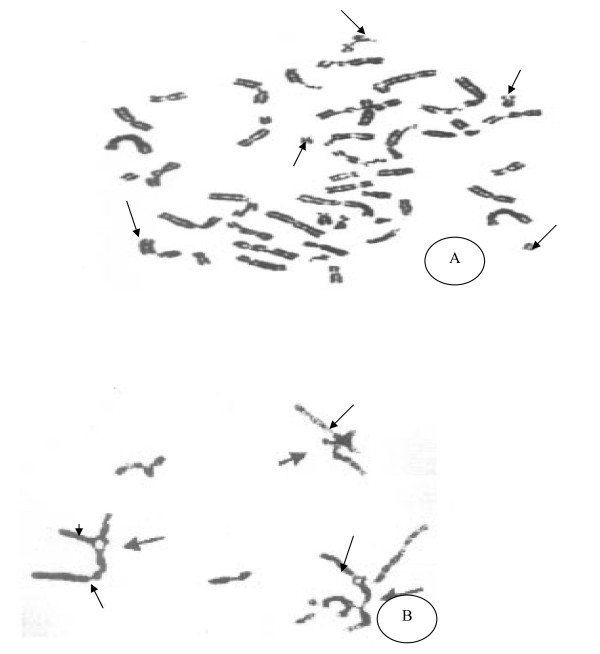
**MMC test metaphase by Giemsa stain**: (A) Increase chromosomal breakage the arrows denoting the area of breakage less than the width of chromatid in FA cases. (B) Radial formation are increased in FA cases.

**- Mitomycin C (MMC) **stress test: induced chromosomal breakage study was carried out on peripheral blood samples or BMA in severe hypoplasia according to a published method [14]. 3-5 ml of whole blood or/B.M was delivered into Green-top (sodium heparin) tube(s). The blood was transferred to a flask containing cell culture media and a cell mitogen, phytohemagglutinin (PHA) for lymphocyte stimulation. The cells are incubated at 37°C for 66 to 72 hours. MMC (40 ng/mg) is added to cultures about 48 hours prior to harvesting the cells for chromosomes studies. Sets of untreated cultures were performed for all the study population samples to serve as a base line, a healthy control samples were done to evaluate the results. In the harvest process, the cells are exposed to colcemid and hypotonic solution, and fixed with glacial acetic acid and methanol. Metaphase cells were dropped onto slides; at least 20 metaphases (stained with Giemsa) were analyzed from each culture (MMC-treated, untreated and healthy control). The MMC-induced chromosomal breakages were then compared to those in the baseline (untreated) culture in all patients groups and healthy control samples. Various chromosome anomalies can be induced with MMC, but in FA, the most specific anomalies are radial configurations and increase breakage. Total radials in 20 cells and the percentage of cells with radials are calculated for the patients groups (hypoplasia, ITP) and the healthy control. Achromatic areas more than a chromosomal width were scored as breaks. The breaks and radial figures were expressed in percent, i.e. number of breaks or radial figures/number of mitotic figures × 100, Figure 2.

- **Diagnosis of hereditary **aplastic anemia (**FA**) was done in Sohag University Hospital, Pediatric and Clinical Pathology Department depending on personal and family history, physical examination, peripheral blood, bone marrow and MMC test.

- **Detection of p53 gene mutation**: was performed in Suiz Canal University Hospital, Clinical Pathology Dept., p53 DNA was extracted from BM samples using the AxyPrep blood genomic DNA Miniprep kit according to manufacturer's instructions (AXYGEN Biosciences, CA, USA). p53 DNA amplification was performed by real-time PCR using TaqMan probe technique in a total volume of 100 μl that included: 10 × Buffer (10 μL), dNTPs Mix 200 nM (2 μL), Forward primer (5'-CCCTGTGCAGCTGTGGGTTG-3') 900 nM (10 μL), Reverse primer (5'-ATGGCCATGGTGCGGAC-3') 900 nM (10 μL), TaqMan Probe (5'-FAM-CCAACCCC CGCCCGGCA-TAMRA-3') 300 nM (10 μL), Hot Start Taq polymerase 2.5 U (0.5 μL), MgCl_2 _100 mM (5 μL), H_2_O (42.5 μL), and p53 DNA template (10 μL). PCR amplification was done in Rotor-Gene™ 6000 real-time rotary analyzer (Corbett Research Pty Ltd, Mortlake, NSW, Australia) using the following protocol: (95°C for 7 min) for one cycle, followed by a three-step program: (96°C for 45 Sec), (56°C for 1 min) and, (72°C for 30 Sec) for 40 cycles. And,(30° for 10 sec) for one cycle. (N.B primers, Taqman probe, and master mix were provided by Bioron GmbH, Germany).

### Statistical analysis

Values were expressed as mean ± S.D and percentages as appropriate. t-test was used for peripheral blood variables.p53 was compared between groups using Fisher exact test.

## Results

Characteristics of the study population are demonstrated in (table 1):

**Table 1:** Study population characteristics

Variables	(FA) patients (n = 10)	(AAA) patients (n = 10)	(ITP) Control (n = 20)
**Age **(yrs) (mean ± SD)	8.8 ± 2.2 **	4.7 ± 2.5	10.5 ± 2.1

**Gender **(M:F)	5:5	4:6	7:13

**Clinical features:**	Skeletal deformity	No specific findings	Purpuric eruption

FA = Fanconi Anemia; AAA = Acquired Aplastic Anemia, ITP = Immune Thrombocytopenic Purpura ** Highly Significant p < 0.001

Regarding the age, FA children are the youngest one at time of presentaion comparing to AAA and ITP (p < 0.001). Sex distribution is equal in FA (50% for both sex), in AAA is (40% males, 60% females), and in ITP the females are predominant (35% males, 65% females).

The peripheral blood and the BM findings are illustrated in (table 2, Figure 1), TLC is highly significant decrease in FA patients and in AAA (p < 0.001). Hb showed a significant decrease in FA and AAA (p < 0.05), MCV is highly significant increase in FA (p < 0.001). Platelets count shows severe thrombocytopenia in ITP, moderate to severe thrombocytopenia in FA and mild thrombocytopenia in AAA. Increase in megakaryocytes is present in marrow aspirates from ITP, hypocellular marrow is found in both FA and AAA samples. BMB demonstrates that, the hypoplasia grade 1,2,3 are present in 8 cases with FA (80%), and in 6 cases with AAA(60%). Marrow fibrosis is present in 3 cases with AAA (30%).

**Table 2:** Peripheral hemogram and bone marrow variables in the studied groups

Variables	(FA) patients (n = 10)	(AAA) patients (n = 10)	(ITP)Control (n = 20)
**Peripheral blood **(mean ± S.D)	2.4 ± 0.6 **	3.3 ± 0.45 **	8.05 ± 0.85
- TLC (× 10^9^/L)	8.4 ± 0.9 *	9.1 ± 0.5 *	11.3 ± 0.55
- Hb (g/dl)	102.4 ± 6.3 **	79.4 ± 3.5	83.3 ± 7.8
-MCV fl	46.1 ± 8.4 **	110.5 ± 12.0	10.7 ± 2.1 **
- Platelets (× 10^9^/L)			

**BMA **	Hypocellular	Hypocellular	Increase megakar -yocytes

**BMB **			Not done
**Cellularity **(no, %)			
- Hypocellular (grades 1, 2, 3)	8/10 (80%)	6/10 (60%)	-
- Normal (grade 4)	2/10 (20%)	1/10(10%)	-
- Hypercellular (grades 5, 6)	-	-	-
**Fibrosis **	-	3/10 (30%)	-

FA = Fanconi Anemia; AAA = Acquired Aplastic Anemia; TLC = Total Leucocytic Count; Hb = Hemoglobin, MCV = Mean Courpascular Volume, * significant p < 0.05, ** Highly Significant p < 0.001

MMC stress test is illustrated in (table 3, Figure 2), where increase in chromosomal breakage and radial chromatin formation are found in FA patients, normal induction time in AAA and ITP comparing to healthy test control.

**Table 3:** Mytomycin C stress test

Variables	(FA) patients (n = 10)	(AAA) patients (n = 10)	(ITP)Control (n = 20)
**Radial formation **	Increased	Normal	Normal

**Chromosomal breakage **	Increased	Normal	Normal

FA: Fanconi Anemia; AAA: Acquired Aplastic Anemia, ITP: Immune Thrombocyt-openic Purpura

P53 gene mutation in the study population (table 4), p53 gene mutation expression was significantly higher in FA (80%) compared to AAA (10%) and control group (0%) (p < 0.001).

**Table 4:** p53 gene expression by real-time PCR

	(FA) patients (n = 10)	(AAA) patients (n = 10)	(ITP) Control (n = 20)
**BM p53 DNA:**			
No. (%)			
Positive	8/10 (90%)	1/10 (10%)	0
Negative	2/10 (10%)	9/10 (90%)	10/10 (100%)

P value*	< 0.001		

FA: Fanconi Anemia; AAA: Acquired Aplastic Anemia, ITP: Immune Thrombocytopenic Purpura

DNA breakage and p53: a positive correlation between DNA breakage and p53 mutation was demonstrated in FA patients (p < 0.02, r0.81).

## Discussion

p53 protein over expression in IBMFS may represent an early indicator of significant DNA genetic alteration, which is a crucial step in the process of leukemogenesis. Thus, up regulation p53 mutation gene in our FA patients may reflect a state of reaction to cell stress mostly related to DNA damage as previously reported [6]. This activation was explained previously by a drastic increase in the half-life of the p53 protein, leading to a quick accumulation of p53 in stressed cells [15].

P53 mutation was not expressed in most AAA patients (90%) and all control group patients (ITP) (100%) as previously described [12]. To ensure that cells under non-stressed conditions (as in AAA and control group) are able to grow, p53 sets up a negative feedback loop by inducing a protein called Mdm2 [16]. Mdm2 is able to both inhibit the transcriptional regulation by p53 [17] and, degrade it thus, maintaining p53 inactive until it is required [18-20].

Expression p 53mutation was significantly up regulated in the marrow samples of FA patients (80%). This probably reflected a functional up-regulation of p53 gene to provide additional protection for cells with damaged and potentially hazardous DNA. Apart from two FA patients who showed no expression of p53 mutation in the BM, a good concordance between p53 mutation gene expression and FA was observed. Of note is that these two patients had moderate DNA breakage points and normal BM cellularity. Despite the small number of patients, which, is a limitation of this study, we conclude that FA in Egyptian children is associated with an up regulation of p53 gene mutation which could explain the tendency of those patients to develop malignancies, on the other hand the AAA, only one case (10%) showed the expression of the mutant p53, this case has a severe hypoplastic marrow which postulate the possible oncogenic cause in AAA. However, future studies on larger population are warranted [21].

## List of Abbreviations and symbols

AAA: Acquired Aplastic Anemia; BM: Bone Marrow; BMA: Bone Marrow Aspiration; BMB: Bone Marrow Biopsy; FA: Fanconi Anemia; Hb: Hemoglobin; ITP: Immune Thrombocytopenic Purpura; MMC: Mytomycin C test; TLC: total leucocytic; PB: peripheral blood; *: Fischer exact test, *: Significant p-value, **: Highly Significant p-value

## Competing interests

The authors declare that they have no competing interests.

## Authors' contributions

All authors read and approved the final manuscript.
